# Single and Synergistic Effects of Fenbendazole and Metronidazole Against Subclinical Infection by *Giardia duodenalis* in Non-Human Primates in a Zoological Garden in Southern Italy

**DOI:** 10.3389/fvets.2022.929443

**Published:** 2022-06-16

**Authors:** Michele Capasso, Lavinia Ciuca, Isabel Guadano Procesi, Francesco Zinno, Federica Berrilli, Giuseppe Cringoli, Laura Rinaldi

**Affiliations:** ^1^Department of Veterinary Medicine and Animal Production, University of Naples Federico II, Naples, Italy; ^2^Zoo Delle Maitine, Naples, Italy; ^3^PhD Program in Evolutionary Biology and Ecology, Department of Biology, University of “Tor Vergata, ” Rome, Italy; ^4^Department of Clinical Sciences and Translational Medicine, Faculty of Medicine, University of Rome “Tor Vergata, ” Rome, Italy

**Keywords:** non-human primates, metronidazole, fenbendazole, zoos, *Giardia duodenalis*

## Abstract

The aim of this study was to assess the single and synergistic effects of fenbendazole (Fenb) and metronidazole (Metro) for the treatment of *Giardia duodenalis* infection in different species of non-human primates (NHPs) housed in a zoological garden of southern Italy. Moreover, the study also aimed to better define the circulation of *G. duodenalis* zoonotic assemblages in NHP and the potential occurrence of zoonotic transmission between the staff from the zoo and NHP. Briefly, six species that belonged to four families (Lemuridae, Cercopithecidae, Atelidae, and Hylobatidae) of NHP and housed in six cages (CG) were identified as *Giardia* positive and divided into two groups. Group F (*N* = 16 animals) was treated with Fenb (50 mg/kg, every 24 h for 5 consecutive days) and Group M (*N* = 7 animals) was treated with Metro (25 mg/kg, two times a day for 5 consecutive days). After the first round of therapy, all the animals were retreated for 5 days by inverting the drugs in each group. On each sampling day [study days (SDs) 3–24], the samples were tested for the presence of *Giardia* cysts using the FLOTAC technique. Multiple fecal tests for the antigen detection of *Giardia*, such as rapid ELISA and direct immunofluorescence (IFA), were performed at each sampling point only on samples that resulted in positive for *Giardia* cysts with FLOTAC. The efficacy of Fenb ranged from 30 to 67% and for Metro ranged from 82 to 96%. The results showed the synergistic effects of Metro and Fenb (98–100%) over the combination of Fenb and Metro (52–90%) against the infection by *Giardia* in NHPs. The overall k agreement between FLOTAC and IFA was reached 0.858 (*p* = 0.0001). In contrast, all the samples had a negative antigen result when using ELISA. At molecular analysis, six samples were confirmed positive for *Giardia* by nested PCR. Only two positive samples were successfully sequenced that showed 100% of identity with assemblage B. All the samples from the humans included in the study resulted in negative for *Giardia* cysts. Overall, the study emphasizes the need for regular monitoring of *Giardia* infections in NHP housed in zoos by traditional diagnostic tools combined with molecular characterization of the parasite.

## Introduction

*Giardia duodenalis* (syn. *Giardia lamblia, Giardia intestinalis*) is a ubiquitous enteric flagellated protozoan of global importance that infects a wide range of hosts, i.e., >40 animal species, such as humans. It is a common leading cause of infection known as giardiosis (or giardiasis) and infects up to ≈ 28.2 million people worldwide, with 500,000 new cases every year (1). *Giardia duodenalis* has been frequently identified as a pathogen in non-human primates (NHPs) (2–6). The prevalence of *G. duodenalis* in NHP that were kept in zoos in different European countries was ranged between 6 and 70% in studies conducted in Belgium (7), Croatia (8), Poland (9), Slovakia (10), Spain, and Brazil (11, 12). A recent study conducted in six European zoological gardens (located in France, Germany, and Spain) reported an 18.1% prevalence of *G. duodenalis* with the presence of both assemblages A and B (6). In Italy, the presence of *G. duodenalis* has been reported in NHP that were kept in the Bioparco in Rome with a prevalence of 47.0% in *Lemur catta* (3), and recently also in NHP were kept in four zoos in central and southern Italy with an overall prevalence of 3.3% (13), whereas the parasite was not reported in captive cynomolgus macaques (*Macaca fascicularis*) imported from registered breeding facilities in China (14). The assemblages of *G. duodenalis* found in the NHP in Italy are mainly A and B, with the dominant assemblage B (3, 15, 16). Giardiosis in NHP causes diarrhea and slow growth, especially in juvenile animals (17). However, few studies have demonstrated an association between the presence of *G. duodenalis* infection and the occurrence of clinical manifestations (18), strongly suggesting that the pathogenic role of *G. duodenalis* in captive NHP is limited (6). Nevertheless, NHPs play an important role as reservoirs of zoonotic *Giardia* infections in the zoological gardens (6, 19–21). The most common therapy used against giardiosis in veterinary medicine is based on metronidazole (Metro) and fenbendazole (Fenb) (22). Metro, such as tinidazole, is used as a single dose in the treatment of giardiosis in humans with high rates of healing (about 90%) and low complications (23, 24). Additionally, tinidazole has been used in the treatment of giardiosis in Barbary macaque (25). Though *Giardia* is a common parasite of zoonotic relevance in NHP, a limited number of field studies have been conducted on the efficacy of antiparasitic treatments in NHPs. Both Fenb and Metro are recommended as therapy for protozoa infections in NHP (26–28). The aim of this study was to evaluate the single and synergistic effects of Fenb and Metro for the treatment of *G. duodenalis* subclinical infection in different species of NHPs that were housed in a zoological garden in southern Italy. Moreover, the study also aimed to better define the circulation of *G. duodenalis* zoonotic assemblages in NHPs and the potential occurrence of zoonotic transmission between the staff (zookeepers and veterinarians) from the zoo and NHPs.

## Materials and Methods

### Animals and Housing

The study was conducted in a zoological garden that is located in the Benevento province of southern Italy (Pesco Sannita, 41°13'57”N; 14°48'40”E). Husbandry in this zoo is based on the European Association of Zoos and Aquaria (EAZA) Best Practice Guidelines for each species or similar, providing the best possible care with good levels of welfare and with sanitary safety for animals, staff, and visitors. Employees and visiting staff working with NHPs wore personal protective equipment when in contact with the animals or their fecal material (13).

This study was performed under the annual preventive medicine plan of the facility recognized by the National/International Regulations for the EU Zoo Directive (DL73/2005 and 92/65 CEE). The Zoo includes a variety of 115 species, housing over 500 animals, and is in continuous expansion. Currently, there are 42 NHPs belonging to 11 species of the following families: Lemuridae, Cercopithecidae, Atelidae, Hylobatidae, and Cebidae. Animals' food consists of specific commercial food combined with daily fresh fruit, vegetables, seeds, eggs, and/or mealworms. Freshwater is provided daily *ad libitum* in polycarbonate water bottles. The cages are cleaned three times a week and disinfected every 2 weeks.

### Parasitological Screening–NHP and Humans (Staff From *Delle Maitine* Zoo)

All the NHPs from the zoo were firstly screened for identification of *G. duodenalis*. Fecal samples (pools) were collected from 11 cages (*N* = 42 subjects of NHPs belonging to 11 species) and subjected to copromicroscopic analysis using the FLOTAC technique as detailed below (29, 30). Six cages (CG) out of 11 (*N* = 23 animals) resulted positive for *Giardia* cysts [mean cyst per gram (CPG) of *Giardia* in each cage: 300 CPG in CG1; 1,200 CPG in CG2; 240 CPG in CG3; 2,280 CPG in CG4; 950 CPG in CG5; and 273 CPG in CPG6]. One of the cages was positive also for *Blastocystis* sp. All the NHPs that resulted positive for *Giardia* cysts were included in the treatment groups. In addition, all the zookeepers (*N* = 4) and veterinarians (*N* = 2) from the zoo were tested for the detection of *Giardia* cysts using the same technique that was used for NHPs (30). All the human stool samples resulted in negative for *Giardia* cysts.

### Laboratory Analysis

Fecal samples analyzed at each sampling point were represented by pools (5–10 g) and collected from the inner core of the cages. Analyses were performed within 24 h of sampling. On each sampling day, the fecal samples were tested for the presence of *Giardia* cysts using the FLOTAC technique based on zinc sulfate (specific gravity = 1.350) flotation solution with a detection limit of 1 CPG of feces (29, 30). Moreover, multiple fecal tests for the antigen detection of *Giardia*, such as rapid enzyme immunoassay (Remel Xpect *Giardia/Cryptosporidium*, Thermo Fisher) and direct immunofluorescence (IFA; MeriFluor *Giardia/Cryptosporidium*, Bioscience), were performed at each point of the study only on samples that resulted positive for *Giardia* cysts with FLOTAC (29).

### Molecular Analysis and Sequencing

Genomic DNA was extracted from six fecal samples of NHPs (pools from each cage) using the QIAamp DNA Stool Mini Kit (Qiagen, Italy) following the instructions of the manufacturer. To identify *G. duodenalis*, the triosephosphate isomerase (TPI) fragment was amplified by nested PCR using the protocol described by Sulaiman et al. (31). Briefly, for the primary PCR, a PCR product of 605 bp was amplified by using primers AL3543 [5′-AAATIATGCCTGCTCGTCG-3′] and AL3546 [5′-CAAACCTTITCCGCAAACC-3′]. For the secondary PCR, a fragment of 530 bp was amplified by using 2.5 μl of primary PCR reaction and primers AL3544 [5′-CCCTTCATCGGIGGTAACTT-3′] and AL3545 [5′-GTGGCCACCACICCCGTGCC-3′]. Molecular analyses were carried out by amplifying an 18S rRNA fragment for *Blastocystis* sp. The specific primer BhRDr (GAGCTTTT-TAACTGCAACAACG) and the broad-specificity eukaryote-specific primer RD5 (ATCTGGTT-GATCCTGCCAGT) were used in a standard PCR reaction with Taq DNA polymerase (BIOTAQ, Bioline, UK) using the protocol described by Stephanie et al. (32).

PCR products were analyzed by agarose gel electrophoresis and visualized after ethidium bromide staining. Subsequently, all the secondary PCR products were sent for sequencing to the Bio-Fab Research, Rome, Italy. Sequences for each amplified region were compared to those previously published in the GenBank database. Identities at the assemblage/subtype level were verified using the Basic Local Alignment Search Tool (BLAST). Sequences were submitted to GenBank under Accession number ON246260-ON246261 for *G. duodenalis* TPI locus and ON215732 for *Blastocystis* 18S rRNA fragment.

### Study Design and Treatment Groups

The study design is summarized in Table 1. Six species that belonged to four families (Lemuridae, Cercopithecidae, Atelidae, and Hylobatidae) of NHP (*N* = 23 animals) and housed in six cages (CG) were identified as *Giardia* positive and divided into two groups: the Group F treated with Fenb (Panacur^®^, 2.5%, Intervet Italia Srl; 50 mg/kg, orally, every 24 h for 5 consecutive days) and the Group M treated with Metro (ERADIA^®^, Virbac Italia Srl; 25 mg/kg, orally, two times a day for 5 consecutive days). After 5 days from the first round of therapy, all the animals were retreated for 5 days by inverting the drugs in each group, as follows: Metro (25 mg/kg, orally, every 24 h for 5 consecutive days) in Group F and Fenb (50 mg/kg, orally, two times day for 5 consecutive days) in Group M. The treatment groups were allocated based on the distance and position of the cages in the zoo where the NHPs were housed according to the species. Specifically, the Group F included three cages housing the following species of NHP each: 12 male dogs of *Lemur catta* (ring-tailed lemur) in CG1; one male and one female of *Cercopithecus mona* (mona monkey) in CG2 and two female dogs of *Alouatta caraya* (black howler) in CG3. Group M included three CG with the following species of NHP: one male and one female of *Nomascus concolor* (black crested gibbon) in CG4; one male and one female of *Colobus Guereza* (mantled guereza) in CG5; and two female dogs and one male of *Semnopithecus entellus* (gray langur) in CG6. For ethical reasons, no untreated control group of animals was available. All the animals were monitored for the presence of *Giardia* cysts and *Giardia* antigen for 24 study days (SDs) as follows: before treatment (SD3–SD1), during the first treatment (T1) (SD2–SD6); during post-treatment-T1 (SD7–SD13); during the second treatment (T2) (SD14–SD18), and post-treatments (SD19–SD24). All the animals included in the study were healthy animals as determined by a physical examination performed before (3–1 days) the beginning of the study. Moreover, all the animals received physical examinations by a veterinarian during the treatments and on the last day of the trial.

**Table 1:** Study design.

Treatment-groups	Group F (Fenbendazole): 50 mg/kg fenbendazole, for 5 consecutive days+metronidazole 25 mg/kg, twice a day for 5 consecutive days Group M (Metronidazole): 25 mg/kg metronidazole, twice a day for 5 consecutive days+50 mg/kg fenbendazole, for 5 consecutive
No. animals per Group	Group F:16 Group M:7
Cages (CG) per Group	Group F: CG1; CG2; CG3 Group M: CG4; CG5; CG6
Study days (SD) and activities	SD-3-SD1(screening for *Giardia* cysts; physical examinations; selection treatment-groups) SD2-SD6 (treatments (T1) fenbendazole/metronidazole -Groups F/M) SD14-SD18 (treatment (T2) metronidazole/fenbendazole-Groups F/M)
Laboratory analysis	SD-3-SD24 Flotation test [FLOTAC technique (29)] Rapid ELISA -Remel Xpect, *Giardia/Cryptosporidium* (Thermo Fisher) Direct immunofluorescent assay (IFA) -Merifluor *Giardia/Cryptosporidium* (Bioscense)

### Drug Administration

The medicated meal was obtained using specific “meatballs” consisting of a moistened mixture of primate-pellets, honey, yogurt, or chopped fruit, which easily allowed the administration of the drugs to the animals. Noteworthy, this type of drug administration allowed the veterinarians to apply the correct amount of the drug for each animal (individually) according to their weight.

### Treatment Efficacy

Treatment efficacy was evaluated based on *Giardia* CPG of feces on SDs 7–12 and SDs 19–24 for both groups (33).


$$\begin{array}{l} \%\;Efficacy\\ \;=\frac{\;Mean\;CPG\;SD\;Pre-T\;Mean\;CPG\;SD\;Post-T}{Mean\;CPG\;SD\;Pre-T}\;\chi \;100 \end{array}$$

CPG = cysts per gram feces.

### Statistical Analysis

Statistical analysis was performed using Windows SPSS^®^ (version 17.0). The non-parametric Mann-Whitney U test was used to determine the level of significant difference between groups of treatment (F and M). Moreover, Kappa (k) statistic was employed to determine the strength of agreement between FLOTAC/IFA and FLOTAC/rapid ELISA, using the following criteria: ≤ 0.2 = poor; 0.21–0.40 = fair; 0.41–0.60 = moderate, 0.61–0.80 = good, and ≥0.80 = very good (34). The level of significance was set at a *p*-value of 0.05.

## Results

The values of *Giardia* CPG in both groups (F and M) and for each SD are shown in Tables 2, 3. Tables 4, 5 show data of mean *Giardia* CPG and efficacies (%) of Fenb and Metro treatments calculated at different post-treatment days. Briefly, in Group F, the results of the parasitological analyses on the SD1 (pre-treatment) revealed a mean value of 2,160 CPG of *Giardia* and on SD 13, after the first treatment, the mean value was 1,257 CPG. In Group M, on SD1, the mean value of *Giardia* CPG was 2,427 and after the first treatment with Metro, on SD 13, the mean value was reduced to 227 CPG. However, after the second treatment in both groups, by using Metro in Group F and Fenb in Group B, the mean of CPG decreased significantly, in particular in Group M (Tables 4, 5). Moreover, the results of the mean CPG of *Giardia* for each cage after 6 days of treatment with every single drug used in the study were the following: post-treatment with Fenb/Metro in Group F (*n* = 261/6 mean CPG in the CG1, *n* = 752/0 mean CPG in the CG2, and *n* = 5,200/945 mean CPG in the CG3) and post-treatment with Metro/Fenb in Group M (*n* = 368/1 mean CPG in the CG4, *n* = 162/15 mean CPG in the CG5, and *n* = 334/11 mean CPG in the CG6). The efficacy of Fenb was ranged from 30 to 67% on SDs 7–12 and from 52 to 90% on SDs 19–24 after the second treatment with Metro. In Group M, the efficacy of Metro ranged between 82 and 96% on SDs 7–12 and 98 and 100% on SDs 19–24 after the second treatment with Fenb. Overall, the synergistic effects of Fenb and Metro against the *Giardia* infection in Group M showed a statistically significant difference (*p* = 0.001) as compared to the Group F. All the subjects included in both groups F and M continued to eliminate *Giardia* cysts after the first treatment with either Fenb or Metro. Instead, all the subjects from the cages CG1 and CG3 remained *Giardia* negative and the only subjects from CG2 remained *Giardia* positive after the second treatment with Metro in Group F. Furthermore, all the subjects from Group M remained *Giardia* negative after the second treatment with Fenb, at the end of the study (SD 24). In addition, the subjects from CG2 and CG6 (*Cercopithecus mona* and *Semnopithecus entellus*) presented co-infections with other parasites, such as *Trichuris* sp. and *Blastocystis* sp.

**Table 2:** Results of *Giardia* cyst per gram (CPG) for all the animals in each cage (CG1, CG2, and CG3) during the entire study (pre-treatment and post-treatments T1 and T2) for Group F.

**Group F**	**T I (Fenbendazole)**												**T II (Metronidazole)**											
	**CPG-days**																							
	**Pre-T**	**T0**					**Post-T**						**Pre-T**	**T0**					**Post-T**					
	**1**	**2**	**3**	**4**	**5**	**6**	**7**	**8**	**9**	**10**	**11**	**12**	**13**	**14**	**15**	**16**	**17**	**18**	**19**	**20**	**21**	**22**	**23**	**24**
CG1	3,600	0	10	0	10	240	416	240	0	72	0	840	600	0	180	72	0	0	0	25	0	12	0	0
CG2	2,400	960	1,200	2,880	9,600	3,800	1,800	4,800	10,000	12,000	1,200	1,400	2,800	1,568	680	2,000	480	890	1,800	460	890	360	600	1560
CG3	480	120	NE	120	240	180	1,200	760	530	0	924	1,100	370	0	10	0	0	0	0	0	0	0	0	0

*T0: the first day of treatment; NE, Not examined; Giardia CPG, the total number of cysts per gram eliminated by all the animals from each cage*.

**Table 3:** Results of *Giardia* cyst per gram (CPG) for all the animals in each cage (CG4, CG5, and CG6) during the entire study (pre-treatment and post-treatments T1 and T2) for Group M.

	**T I (Metronidazole)**												**T II (Fenbendazole)**											
	**CPG-days**																							
	**Pre-T**	**T0**					**Post-T**						**Pre-T**	**T0**					**Post-T**					
	**1**	**2**	**3**	**4**	**5**	**6**	**7**	**8**	**9**	**10**	**11**	**12**	**13**	**14**	**15**	**16**	**17**	**18**	**19**	**20**	**21**	**22**	**23**	**24**
CG4	4,560	1,600	480	120	60	10	270	96	720	84	498	538	300	418	294	174	70	0	0	8	0	0	0	0
CG5	1,900	1,200	600	240	0	0	4	30	0	360	250	330	250	340	366	228	132	54	32	50	6	0	0	0
CG6	820	400	220	72	140	560	840	120	240	0	376	426	130	290	412	314	98	20	0	18	40	10	0	0

*T0: the first day of treatment; Giardia CPG: the total number of cysts per gram eliminated by all the animals from each cage*.

**Table 4:** Results of mean *Giardia* cyst per gram (CPG) and efficacy (%) of the treatments performed in Group F, on study days (SDs) 7–12 and SDs 19–20.

**Group F**
**Pre-T (mean CPG = 2,160)**
	**Mean CPG**	**% Efficacy**
**Study days post-T (T1) Fenbendazole**		
7	1,500	30.6
8	1,933	11.7
9	3,510	0
10	4,024	0
11	708	67.2
12	1,113	48.5
**Pre-T (mean CPG** **=** **1,257)**
**Study days post-T (T2) metronidazole**	
19	600	52.2
20	162	87.1
21	297	76.4
22	124	90.1
23	200	84.1
24	520	58.6

**Table 5:** Results of mean *Giardia* cyst per gram (CPG) and efficacy (%) of the treatments performed in Group M, on study days (SDs) 7–12 and SDs 19–24.

**Group M**
**Pre-T (mean CPG** **=** **2,427)**
	**Mean CPG**	**% Efficacy**
**Study days post-T (T1)** **metronidazole**	
7	371	84.7
8	82	96.6
9	320	86,8
10	148	93.9
11	374	84.6
12	431	82.2
**Pre-T (mean CPG** **=** **227)**
**Study days post-T (T2) fenbendazole**	
19	11	95.1
20	25	89
21	15	93.4
22	0	98.7
23	0	100
24	0	100

Moreover, all the fecal samples from each sampling point resulted in negative at the antigen detection of *Giardia* using the rapid enzyme immunoassay when compared with direct IFA, which resulted in all positive. The overall k agreement between FLOTAC and IFA was 0.858 (*p* = 0.0001). Analyses were not carried out when comparing FLOTAC with the rapid ELISA, given that no sample resulted positive with the ELISA test.

All six samples were confirmed positive for *Giardia* by nested PCR. Only two positive samples (CG1 and CG2) were successfully sequenced that showed 100% of identity (498/498; 100% query coverage) with assemblage B (Accession number: MF095053). *Cercopithecus mona* from CG2 also resulted positive for *Blastocystis* sp.; the sample was successfully sequenced showing 100% of identity (579/579; 100% query coverage) with Subtype ST1 (Accession number: MN338073).

## Discussion

Little is known about the occurrence and genetic diversity of *Giardia duodenalis* in NHPs; however, giardiosis has been described in the following species of NHPs: squirrel monkeys, rhesus macaques, lemuridae, and marmosets (3, 25, 35–37).

Though *Giardia* is a common parasite in zoo-housed primates, a few studies have evaluated the treatment options for giardiosis in NHP. One study reported the efficacy of tinidazole in Barbary macaque (25) and another study assessed whether oral administration of Metro dissolved in drinking water would be successful to eliminate *Giardia* cysts in rhesus macaques (38). Our field study evaluated the efficacy of Metro and Fenb and assessed the synergistic effect of the two drugs against *G. duodenalis* infection in different species of NHP housed in a zoological garden in southern Italy. This study revealed a high and persistent detection of *Giardia* cysts in various species of NHPs that include *Lemur catta, Cercopithecus mona, Alouatta caraya, Nomascus concolor, Colobus guereza*, and *Semnopithecus entellus*. These results obtained are consistent with other similar studies (3, 7, 8, 11). The occurrence of *Giardia* was reported in NPH species that were not well-documented until now, such as mona monkeys, black howler, black-crested gibbon, mantled guereza, and gray langur.

The results on the efficacy of Fenb vs. Metro and their synergistic effect on *Giardia* infection underlined that almost all the animals became negative when combining Fenb at 50 mg/kg sid for 5 days with Metro at 25 mg/kg bid for 5 days. The only group of animals that remained positive for *Giardia* cysts at the end of the study was the one that belong to the Cercopithecidae family (the pair of one female and male of mona monkeys). In addition, they had the highest infection rate due to the variation in cyst shedding per day. Our data further indicate that pooling serial fecal samples from multiple colony animals likely would identify the presence of *Giardia* in a colony.

In the present study, *Giardia* infection in NHPs was not associated with clinical signs of diarrhea. These findings contrast with several studies on humans, in which 60–70% of infected persons show signs of diarrhea (39). Instead, the condition observed in the NHPs seems to be similar to the chronic asymptomatic carrier state that occurs in companion animals (40–43). This condition of subclinical *Giardia* infection indicates that NHPs may represent important reservoirs and serve as a source of zoonotic infection for other animals.

This study provided an unexpected finding concerning the diagnostic tools for antigen detection of *Giardia*. In fact, all the samples that resulted positive with FLOTAC were confirmed with the gold standard IFA, assessing a very high agreement between the two techniques (30). However, the present study yielded discordant results when using the rapid test (Remel *Giardia/Cryptosporidium*) on *Giardia*-positive samples. In fact, all the fecal samples from each point of the study that were positive at FLOTAC and IFA had a negative antigen result with the rapid test used. These findings are not in agreement with several studies that reported the rapid enzyme immunoassays as a precise tool for detecting *Giardia* in fecal specimens with test sensitivities and specificities that have approached 100% (44–46). However, future work should include investigations regarding the combined factors that may result in *Giardia*-antigenic stimulation of the intestinal tract in NHP.

The molecular analysis revealed that assemblages A and B in all samples tested positive for *Giardia*-DNA. *Giardia* zoonotic assemblages A and B have been already described in NHP in many studies that include some species of NHPs from a zoological garden in Rome, Italy (3, 47–50). The potential zoonotic *G. duodenalis* assemblage B was identified in two groups of NHP (*Lemur catta* and *Cercopithecus mona*). These findings suggest that ring-tailed lemurs, as already has been reported, and mona-monkeys may be asymptomatic carriers of *G. duodenalis* and a higher parasitic load might occur in these species of NHP held in walk-through enclosures (4). Moreover, all the animals (*Cercopithecus mona*) from CG2 were the only animals that remained positive for *Giardia*, until the end of the study, after two treatments of 5 days each with Fenb and Metro. We cannot rule out reinfection, due to the prepatent period of *Giardia*, that can be as short as 5 days, but on the other side, the host susceptibility of the NHPs for *Giardia* infection can be as well incriminated for the persistence of the *Giardia* cysts. However, the increase in the number of *Giardia* cysts shed by the two animals from CG2, after the treatment, e.g., Day 9 (*N* = 10,000 CPG) and Day 10 (*N* = 12,000 CPG) has occurred in other studies as well (41, 51). It is important to note that the shedding of the mean CPG of *Giardia* from CG2 in Group F resulted in a high value (*N* = 5,200 CPG) post-treatment with Fenb when compared with a low value (*N* = 945 CPG) post-treatment with Metro in the same group.

In addition to the presence of *G. duodenalis*, the other parasites identified in this study were *Trichuris* sp. and *Blastocystis* sp. that could cause serious gastrointestinal enteritis in NHPs (6, 35, 36).

To our knowledge, this is the first study that showed the efficacy of Fenb that ranged from 30 to 67% on SDs 7–12 and the efficacy of Metro that ranged between 82 and 96% on SDs 7–12 against the subclinical infection by *Giardia* in NHPs. Moreover, the final output revealed the synergistic effects of Metro and Fenb (98–100%) over the combination of Fenb and Metro (52–90%) against the infection by *Giardia* in NHPs. In addition, tinidazole can be a good therapeutic strategy for giardiosis in NHPs, as it has been already demonstrated in marmosets (25); however, future work should include the evaluation of the efficacy of this drug in other species of NHP. Indeed, the evaluation of health profile markers of pre- and post-treated animals would have underlined the safety of the drugs used in this study.

The results of this study showed that *G. duodenalis* is a common parasite in NHP in southern Italy. The findings of this study enriched the list of host species susceptibility of NHPs to *G. duodenalis* from Italy, by adding other species of NHPs, such as *Cercopithecus mona, Nomascus concolor, Colobus guereza, Semnopithecus entellus*, and *Alouatta caraya*, that were either not studied or tested negative for *Giardia* cysts in previous studies. Furthermore, the substantial variation in the number of *Giardia* cysts per gram shed on various days of the study could be explained by the differences in susceptibility in the host species of NHPs or by the continuous exposure to cysts from the environment. Indeed, a more effective sampling strategy would have improved the overall output of the study regarding the epidemiological data obtained, for example, collecting individual fecal samples and not pooled samples from the cages as performed in the present study.

For *Blastocystis* sp. detection, as reported by Köster et al. (6), ST1 in NHPs in zoological gardens was uniquely documented in Spain, despite being ST1 the most prevalent genotype worldwide. This trend may be justified by the lack of molecular data regarding *Blastocystis* sp. isolated from zoological institutes (6).

Zookeepers and veterinarian staff resulted negative to the parasitological exams. However, the monitoring of a potential zoonotic transmission must continue to be a priority, possibly with a temporal continuity in sampling and through an increased number of recovered samples. This would enable to assess any potential transmission risk between staff, visitors, and NHPs.

Overall, the study emphasizes the need for traditional diagnostic tools combined with molecular characterization of the parasite and for the elimination of the infection by using an association of Metro and Fenb, in particular, for the NHPs that are asymptomatic with marked persistence of cysts, in order to better assess and reduce the zoonotic risk of *Giardia* infection. Regarding the detected zoonotic assemblages in the NHP from the Maitine Zoo in southern Italy, NHPs are considered as important hosts of *G. duodenalis*; it is therefore strongly recommended to conduct the screening of this infection within the routine examinations in zoological gardens all over the world.

## Data Availability Statement

The datasets presented in this study can be found in online repositories. The names of the repository/repositories and accession number(s) can be found at: https://www.ncbi.nlm.nih.gov/genbank/, MF095053; https://www.ncbi.nlm.nih.gov/genbank/, MN338073.

## Ethics Statement

Ethical approval was not provided for this study on human participants because since the study was carried out on stool samples provided voluntarily by the staff from the zoo (zookeepers and veterinarians), no ethical approval was required for our study. Written informed consent for participation was not required for this study in accordance with the national legislation and the institutional requirements. This study was performed under the annual preventive medicine plan of the facility recognized by the National/International Regulations for the EU Zoo Directive (DL73/2005 and 92/65 CEE).

## Author Contributions

LC, LR, and MC contributed to the conception and design of the study. LC and IP performed the laboratory analysis. IP, FZ, and FB organized the database and performed the statistical analysis. MC, LC, GC, FB, and LR wrote the manuscript. All authors contributed to manuscript revision, read, and approved the submitted version.

## Conflict of Interest

The authors declare that the research was conducted in the absence of any commercial or financial relationships that could be construed as a potential conflict of interest.

## Publisher's Note

All claims expressed in this article are solely those of the authors and do not necessarily represent those of their affiliated organizations, or those of the publisher, the editors and the reviewers. Any product that may be evaluated in this article, or claim that may be made by its manufacturer, is not guaranteed or endorsed by the publisher.
